# Culturally informed and flexible family based treatment for adolescents: enhancements to better serve adolescents with self-harm behavior

**DOI:** 10.3389/frcha.2025.1516782

**Published:** 2025-10-27

**Authors:** Maite P. Mena, Gabrielle M. del Rey, Melissa A. Gutierrez, Karina A. Gattamorta, Rebecca A. Lazarus, Daniel A. Santisteban

**Affiliations:** ^1^ Department of Educational and Psychological Studies, University of Miami School of Education and Human Development, Coral Gables, FL, United States; ^2^ Training and Implementation Associates, Miami, FL, United States

**Keywords:** family therapy, adolescent, culture, self-harm and suicide related behavior, CIFFTA, evidence based treatment

## Abstract

Culturally Informed and Flexible Family Based Treatment for Adolescents (CIFFTA) is a manualized treatment that has been shown to reduce youth substance misuse and a variety of behavior problems but it has not been used to treat self-harm behavior in youth. The purpose of this manuscript is to describe enhancements that can address the treatment needs of diverse youth reporting suicide risk, cutting, and other self-harming behavior. We describe the enhancements to psychoeducational, individual therapy and family therapy content and processes as well as technological enhancements to improve access to treatment and to engage the adolescent in therapeutic work between therapy sessions. We believe that to help reduce barriers to service utilization in diverse populations, the treatment must also be ecologically valid. We present the case of a 15 year old Latine female who received treatment for cutting behavior and demonstrate CIFFTA's components in action. As we have reported separately, the acceptability of this enhanced intervention is supported by data showing that 93% of the youth and families attended at least 8 sessions and that on average they received over 23 sessions of treatment. This treatment enhancement effort resulted in new tools that were integrated into the manualized CIFFTA making it easier to engage families and deliver interventions. These enhancements culminated in an adaptive, replicable, and culturally informed treatment for diverse youth reporting self-harm and their families.

## Introduction

Youth suicide risk and self-harm behaviors are reported at alarming rates and present complex clinical challenges in treatment (1). Suicide is the second leading cause of death in youth ages 10–24 (2) and male adolescents' complete suicide at four times the rate compared to females (3). And yet, this profile tends to differ considerably from the more prominent profiles of self-harm behavior seen in therapy practices. This is because females show self-harm behavior at a rate of 2.6 to 1 when compared to males (4) and because self-harm behavior includes not only nonfatal suicide attempts (SA) but also non-suicidal self-injury (NSSI).

Regardless of intent, all types of self-harm behavior impede adolescent development and constitute risk for future suicide (5–7). In young people who do not have the motivation or intent to end their lives, self-harm behavior may be a way of coping with emotional distress. Youth with and without the intention to die may have different triggers, intentions, and motivations for the self-injury, but both groups are at higher risk for eventual suicide (8).

## Unique stressors and protective factors that impact self-harm

Latine, Black, and LGBTQ+ youth report particularly high rates of self-harm and related symptoms (9). For many years Latine female adolescents (Ages 14–18) were reported to be 1.5–2 times more likely than non-Latine White and African American peers to consider, plan, or attempt suicide (10–13). This led to an impressive literature documenting risk process in Latine female adolescents (14–17).

More recently, data showed that Black adolescents had surpassed Latine adolescents in the prevalence of suicide attempts with the 2019 YRBS reporting that 11.8% of Black adolescents and 8.9% of Latine adolescents attempted suicide during the previous year (18). Price & Khubchandani (19) reported that “the rate of African American male suicides increased by 60% and for African American females increased by 182% from 2001 to 2017” (p. 756). A 2024 Center for Disease Control report documented a similar profile for the two populations with 10% of Black youth and 11% of Latine youth attempting suicide during the previous year (20).

LGBTQ+ youth are also at increased risk for suicidality (21). Surveys show that 41% of LGBTQ+ youth had seriously considered attempting suicide, compared to 13% of cisgender/heterosexual peers and that 20% of LGBTQ+ youth attempted suicide during the past year (9). Compared with White LGBTQ+ youth, Latine LGBTQ+ youth were more likely to attempt suicide (22). Mueller et al. (23) found that “Black LGB youths were more likely than their White heterosexual same-gender peers to report suicide ideation” (p. 983).

An understanding of *Minority Stress Theory* is helpful in describing how excess stress that comes from belonging to a stigmatized social category or minoritized position can contribute to self-harm. This stress is attributable to more than an individual deficit and is found in the interactions that emerge in unwelcoming social and political contexts. The minority person experiences additional forms of stress as a result of social structures and norms that do not reflect the worldview, beliefs and expectations of the minority group (24, 25). As family therapists, we also appreciate that it is not only the adolescent that is confronting these types of stressors. When caregivers and other family members are being bombarded by these same stressors, their ability to support and buffer the youth is diminished.

Given the rates of self-harm, adverse events, and unique stressors (e.g., racism, discrimination, and marginalization) that have been documented in the lives of Black and Latine youth, their persistent pattern of underutilization of mental health service is particularly problematic. Compared to White youth ages 5–17, in which 14.1% of youth had received counseling or therapy services, only 8.8% of Black and 8.1% of Latines had received such services (CDC, 2023). Minoritized youth and families tend to have fewer office visits for mental health and behavioral presenting problems, less access to evidence based treatments, and often receive services that are not culturally informed or designed to address their unique stressors and needs (26). Youth who are undocumented or who are children of undocumented adults face additional barriers to accessing care, including ineligibility to Medicaid, anti-immigrant laws, and fear of deportation (27). Factors contributing to underutilization of services include socio-political, economic, and culture-related factors (such as stigma), as well as accessibility issues (such as identification of appropriate services, transportation, presence of immigration enforcement officers) (28). Quality services are often lacking due to language and cultural incongruence in mainstream healthcare settings (28). Approaching the presenting problem of self-harm using evidence-based, tailored, and culturally informed treatment may provide a way of increasing service utilization, reducing disparities, and protecting youth from greater suicide risk.

## Promising approaches to treatment

A review of youth self-harm interventions concluded that one factor that may contribute to efficacious treatments is family involvement (29). Family interventions begin with effective engagement strategies (30) to better address normative reluctance to enter family services and the stigma and defensiveness that often comes with learning that a child or adolescent is self-harming and/or contemplating suicide. As therapists seek to engage these families, there must be an understanding that families often hope that self-harm behavior or suicide ideation is a passing phase. Successful engagement of family members and working together toward the goal of keeping the adolescent safe makes treatment more efficient and productive. Family processes such as family connectedness, support, validation, and the ability to resolve conflict are key to successful family work and suicide prevention (31).

A second approach to reducing healthcare access disparities in diverse families is to design and utilize *evidence based and culturally centered* treatments. Treatments that significantly integrate culture-related material have been associated with increased utilization and superior outcomes in diverse populations (32, 33). Treatments that take a bottom-up approach in their design and are most responsive to cultural context and culture-specific concerns appear to drive this effect when compared to treatments that take a top-down approach and make more limited adaptations to existing treatments (32). The bridging and integration of established change processes and culturally informed care can be achieved in manualized and evidence-based treatments (34, 35) and have been shown to be particularly efficacious when used with families that describe themselves as low on acculturation (36). An ecologically valid treatment (37) that addresses unique culture-related stressors (e.g., marginalization, family rejection, racism, and acculturation stress) (37, 38) promises better engagement, service utilization, and reduction of self-harm and suicide among minoritized youth and families.

Finally, the integration of technology has the potential to reduce treatment delivery challenges, provide variable information formats (e.g., multi-media) that are engaging, and encourage therapeutic work outside of formal session times. Research has shown promising results in reduction of self-harm using computer-assisted interventions (40) and especially hybrid treatment combining technology and therapist support (41–43). COVID-19 disruptions to face-to-face meetings accelerated the acceptance of technology-assisted treatment, the identification of advantages/opportunities available in virtual sessions, and the efforts to address its barriers to implementation (e.g., handling of sensitive issues and security of communications) (44).

## Overview of culturally informed and flexible family based treatment for adolescents (CIFFTA) and its enhanced components

**Figure 1:**
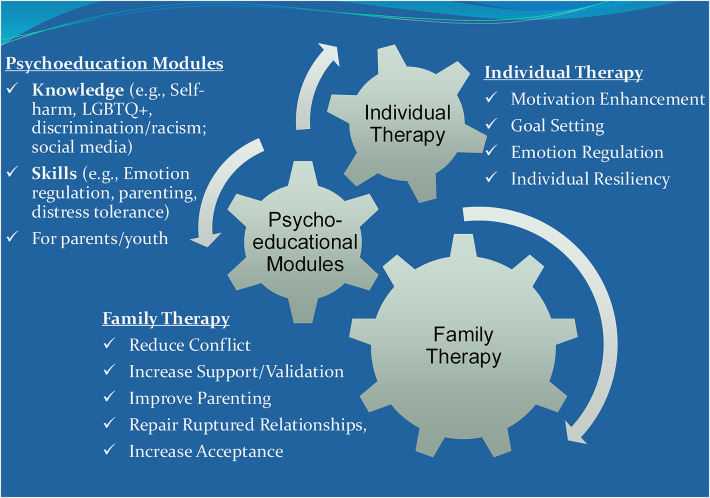
Each CIFFTA component creates change in the other (synergy).

Culturally Informed and Flexible Family Based Treatment for Adolescents (CIFFTA) is a multi-component treatment that delivers *adolescent therapy sessions*, *conjoint family therapy sessions*, and *psychoeducational module sessions* (34, 35, 45). It is a flexible manualized treatment with specific procedures and resources that facilitate the systematic tailoring of treatment components to the unique youth and family needs while remaining replicable. CIFFTA's modular approach offers content options that facilitates the tailoring of the treatment to the unique culture-related (e.g., immigration and acculturation experiences and minority-related stressors), clinical (e.g., depression, substance use, and emotion dysregulation) and family (e.g., blended families and families involved with Juvenile Justice) characteristics (See Figure 1). CIFFTA has been shown to be efficacious in the treatment of adolescent substance misuse (46), a variety of youth behavioral and emotional disorders (36, 47) and in a community-based evaluation (48). In these studies, CIFFTA has been effective at improving family functioning including improving parenting practices and cohesion and reducing family conflict. As an *ecologically valid* treatment that was designed to take real life stressors and cultural factors into account, CIFFTA is considered relevant, timely, and useful by minoritized families.

CIFFTA takes a *transdiagnostic* approach that allows it to address many different symptoms by making systematic adjustments for pre-specified conditions (49). Using this approach means addressing “maintaining mechanisms” (e.g., family functioning and emotion dysregulation) that may underlie several of the co-occurring disorders (50). This may be particularly important in the treatment of self-harm behavior because it is commonly found to co-occur with other mental health (e.g., depression and emotion dysregulation), behavioral (e.g., alcohol and substance misuse), and family (e.g., invalidation and poor communication) presenting problems. The identification of these presenting issues can lead to the use of specific CIFFTA psychoeducational modules in a systematic and replicable fashion. Transdiagnostic treatments may be more acceptable to treatment agencies and clinicians because they can focus on factors that trigger and maintain several different and co-occurring presenting problems rather than focus narrowly on one symptom at a time (49). Although CIFFTA has been tested to address a diverse set of symptoms, it *has not included a specific focus on self-harm behavior and its clinical and family correlates*.

The purpose of this manuscript is to present the enhancements created and integrated to increase CIFFTA's clinical specificity and efficacy with diverse (Latine, Black, and LGBTQ+) youth reporting self-harm behavior and their families. CIFFTA is a manualized treatment, and the systematic integration of self-harm related content was key to building an evidence based and replicable treatment. In the remainder of this manuscript, we present the enhancements created in: (a) psychoeducational content and processes including the integration of technology, (b) individual therapy interventions and processes, and (c) family therapy interventions and processes. We also present guidelines that we developed for the virtual delivery of CIFFTA and a case study that shows the new CIFFTA content and processes in action. We hope that this article can demonstrate how the integration of a modular approach, culturally and clinically focused material, and technology can result in a manualized and replicable treatment for clinicians working with diverse youth reporting self-harm and suicide-related behavior.

## Enhancement of the psychoeducational modules component

CIFFTA includes approximately 20 psychoeducational modules that are designed to provide content in a structured and systematic fashion. Modules provide foundational knowledge about specific topics and issues (e.g., self-harm and impact of substance use), they can normalize certain family experiences (e.g., parent-child separations and blended families) and they can provide strategies and skills that address presenting problems (e.g., depression or emotion dysregulation). They can facilitate behavioral changes sought in individual and family therapy sessions by showing the value of improvements in family engagement, communication, and connection. The selection of the 3–4 most relevant modules from a comprehensive list of 20 modules and the use of a collaborative and shared decision-making process, are key to the CIFFTA tailoring process. Some modules are designed for the family members only (e.g., parenting practices) or the adolescent alone (e.g., emotion regulation) while most others can be delivered to the entire family together.

### Self-harm

A first step to enhancing CIFFTA was the integration of psychoeducational content focused on the distinction between suicide related attempts, cutting, and other self-harm behavior that was not linked to ending life (51). It is important to help adolescents and parents understand the motivations and triggers that may be present and their differing treatment implications (e.g., finding avenues for dealing with emotional pain for cutting behavior vs. finding reasons for living when there is intent to suicide). It was also important to talk about how common suicide ideation and some self-harm-related behaviors are, what types of co-occurring issues place the youth most at risk (e.g., emotion dysregulation, substance use, depression, isolation, and previous suicide attempts (51), and to learn to think about intrapersonal vs. interpersonal triggers (e.g., family argument, bullying (52). Other important content areas included fear of discussing self-harm and the stigma, defensiveness, and denial that can be associated with self-harm.

When we began to work with youth and families around the topic of self-harm, the processes that we had identified in the literature came to life. We encountered families that wanted to put a past self-harm related event behind them as quickly as possible in the hope that it was a one-time event. They often hoped that a brief crisis intervention was sufficient to reduce long-term risk and distress. Other families found it difficult to *air dirty family laundry out in public* or expose behaviors and emotions they felt might bring shame to the family. It became apparent that instilling hope and creating a safe space was essential if a therapist wanted the family to address the difficulties directly. The psychoeducational material was designed to normalize family member reactions to self-harm behavior. The module material highlights the value of discussing these difficult issues and shows how the crisis resulting from the self-harm behavior can become an opportunity for change and growth.

### LGBTQ+ youth and their families

Given the high proportion of LGBTQ+ youth reporting self-harm, we identified many new and important LGBTQ+-related topics to integrate into CIFFTA. At the individual level, we incorporated basic education and training for therapists on mental health and well-being concerns related to rejection, discrimination, and stigma around gender and orientation stereotypes (53). This module prepares the therapist to have a respectful, supportive, affirming, and open discussion with the adolescent. The new content opened the door for conversations about the identity exploration the adolescent may seek to initiate. Because CIFFTA has a strong family component, there was a focus on families who had recently learned about their child's sexual orientation or gender identity and were having difficulty with acceptance (54). We integrated the discussion of the role of culture and upbringing and its influence on their reactions and responses to the youth. We developed content that could be shared with parents and in collaboration with a group of experts on LGBTQ+ services, we integrated reactions and responses from young adults and family members who had gone through the “coming out” process with differing levels of ease/difficulty. From this collaboration, we developed recordings of parents and young adults who shared why it was difficult for them at the start and how they came to realize the importance of openly supporting and loving their children. The delivery of these recordings was facilitated by the technology enhancements discussed later.

The literature points to the importance of preparing therapists to consider and discuss disclosure with youth. Care must be taken when considering the possible costs and benefits of disclosure by adolescents who have not disclosed to parents or who have disclosed only to some family members (55). By incorporating this material into our modules, CIFFTA therapists had up-to-date information and talking points regarding the pros and cons of disclosure, and how the quality of the relationship can determine the family member's response. It should not be assumed that immediate disclosure is the best route in all circumstances, and this is an important decision point for the youth.

### Adolescent skills modules

In focusing on self-harm, the authors’ previous work on Dialectical Behavior Therapy (DBT) skills for youth was particularly useful (56–58). A dialectical behavioral therapy lens prepared therapists to address self-harm resulting from strong surges of emotions that are difficult to tolerate, or from a maladaptive and unsafe response to those emotions (59). Key skills help youth to tolerate emotions without harming themselves or others, to be present in the moment, to manage emotional swings, and to handle relationships more effectively. The core DBT skills reintegrated into CIFFTA included Distress Tolerance, Emotion Regulation, and Interpersonal Effectiveness (60). To introduce the content in a non-threatening and entertaining way, four short and engaging videos (e.g., using PowToon's) were developed that presented the material using graphics and commonplace language. The videos delivered complex information in a standardized manner that could be accessed through computers or phones. The multimedia materials facilitated the attitudinal, emotional, and behavioral changes sought within individual and family therapy sessions. Electronic diary cards were also added that could be used throughout the week to document and track triggers, risk situations, and the skills that may be helpful in those situations. When used within an adaptive framework such as CIFFTA's, these stand-alone modules could be selected if and only if the theme of the module was relevant to the youth and family. As with many skills, the challenge for the therapist was to facilitate the *generalization* of the skills to daily life. While the didactic module sessions helped the adolescent learn new skills, the individual and family therapy sessions became the contexts in which generalization was achieved. The diary card was a useful tool to facilitate generalization.

### Technology to enhance services delivery

The platform developed for CIFTTA had several important features that facilitated and enhanced the impact of the services. Therapists could assign videos to clients depending on the specific needs that the family identified. The language of the video (i.e., whether in English or Spanish) was also the client's choice. The video could be made available remotely by the therapist allowing adjustments to be made immediately. Typically, adolescents and parents were separately assigned 3–4 modules based on their needs and interests. This video selection process supported the tailored/adaptive approach that is a CIFFTA innovation.

#### Interactive nature of videos that inform the subsequent session

In order to build some level of interactivity into the technology, we designed the system so that participants would watch videos and then answer questions about symptoms or concerns (e.g., by clicking on symptoms or concerns). The subsequent treatment session was informed by the participants' questions, responses, and concerns. For example, when watching a video on depression, the parent and/or youth could click on the symptoms the youth was experiencing, (e.g., low energy, disruption in sleep) the information was sent to the therapist, and it was available as the topic of the next therapy session. An additional question asked, “which of these topics would you like to discuss with the counselor?” By merely clicking “yes” or “no” to each query, the stage was set for the next session. The therapist could prepare to address real and pressing adolescent and parent concerns in the next face-to-face session. During the next session, family members would often report to the therapist that the session was responsive to the needs and preferences they communicated while interacting with the system.

#### Diary cards

Therapists valued the use of diary cards to help identify urges and triggers to self-harm (60), so the online platform was designed to allow adolescents to access the diary card from home or from their smartphones, while their experiences were fresh. All the information entered was transmitted to the therapist. If an adolescent forgot to complete the diary card or if something being discussed in an individual or skills session with the therapist triggered an emotion, the online diary card could also be completed in the session. Although not all youth and therapists chose to use the diary cards, the electronic access mitigated some of the barriers for those who did wish to use them.

## Enhancement of the individual adolescent therapy component

Our individual work with adolescents typically focuses on engagement, motivation enhancement, identification of goals, preparation for difficult family interactions, generalization of skills, and monitoring the trajectory of presenting problems (e.g., self-harm, substance use). Because there is evidence of better outcomes for treatments that systematically address group-specific risk and protective factors (61), we integrated work on the identification and modification of unique risk factors and stressors that could be associated with self-harm. In the individual treatment of Latine youth, we helped the adolescent to understand the way acculturation gap stress, discrimination stress, family stress around drug use, and immigration stress can contribute to self-harm behavior (62). As we worked individually to weaken the link between these stressors and urges to self-harm, we also addressed these stressors in the family context. For Latine females specifically, we integrated work by Zayas et al. (63) that showed how Latine females can find themselves in a developmental struggle between wanting to honor the family (e.g., familism) and wanting autonomy. The material focused on helping the adolescents to find more healthy and adaptive ways of differentiation, even in the context of culture-related tensions and their desire for family harmony.

To work more effectively with Black adolescents, therapists were prepared to address culture related stressors for Black youth such as perceived discrimination, hopelessness, and substance abuse all of which have been hypothesized to increase risk for self-harm and suicide (64–66). The literature showed that higher perceived discrimination was associated with higher odds of suicidal ideation, regardless of age, ethnicity, gender, and socioeconomic status (64). Although it helps for therapists to be aware of unique stressors that may emerge, they must also be careful not to assume that all the stressors are present because of the client's race or ethnicity. The therapist must approach families with cultural humility and curiosity and be prepared to listen and learn. The therapist must also be prepared to comfortably engage in the often sensitive and difficult discussions surrounding racism and discrimination (67).

CIFFTA's enhancements prepared therapists to discuss how LGBTQ+ youth face a unique set of stressors that can contribute to self-harm behavior. An integral part of our work was the use of *Minority Stress Theory* to describe how belonging to a stigmatized social category or minoritized position can lead to excess stress and contribute to self-harm (68). Youth were helped to see the power of these sources of stress and how they can learn to buffer themselves from the deleterious effects. This also included how family and peer rejection can be major contributing factors (69). In all such discussions, we acknowledged that while the LGBTQ+ umbrella term was helpful, there is much variation within the umbrella term. For example, the stressors and barriers to acceptance faced by transgender youth may be vastly different than those that sexual minority youth must confront. In our individual work with youth, we explored how long it took the youth to accept perceived changes in themselves and used that experience to help them understand the time that even loving family members might need to process the changes communicated by the youth.

We integrated material showing how minority status in the areas of race, ethnicity, and sexuality can be deeply interconnected targets of oppression, rather than distinct experiences (70). The material emphasized how LGBTQ+ youth of color can experience homophobia, transphobia, and racism within the LGBTQ+ or POC community (71). LGBTQ+ Latine youth may experience more difficulties in the process of coming out, in receiving parental and family support, and the extent to which they feel a sense of belonging in their community when compared to non-Latine White LGBTQ+ youth (72). The high endorsement of religiosity and faith among Latine and Black families, often found to be a protective factor, can also make acceptance of an LGBTQ+ identity more difficult and initial family rejection more likely (71). Conversely, the material highlighted how “a family's ability to understand and respond appropriately and positively to their LGBTQ + child may be one of the strongest protective factors for LGBTQ+ youth and an asset to their successful transition to adulthood” [(69); p. 43]. Treatment that actively engages family members and fosters movement towards family validation and acceptance may be particularly effective at reducing risk and increasing well-being.

Individual treatment is used to explore how race and ethnicity related protective factors and stressors such as gender roles, discrimination, racial identity, acculturation, family factors, and stigma are connected to mental health. For example, the youth's family may be highly family-oriented and value strong family relationships. However, when youth experience negativity, rejection, and lack of verbal affirmations from caregivers (73), sometimes due to acculturation and gender role tensions (17), and sometimes due to sexual or gender orientation, the rejection from the family can feel particularly powerful and painful. In individual treatment youth explore with the therapist the types of family characteristics (e.g., traditionalism, religiosity) that are at times protective but that can also make acceptance more difficult and complex. Sometimes it just means that the adolescent realizes that it might take more time and not that it means that family members will never be capable of acceptance and validation. The videos that were offered to families in our program served to increase the readiness of parents and youth to discuss the complexity of the situation. Parents learned to accept their youth in a deeper way and were helped by hearing the stories of other families with similar experiences and emotions.

For youth and family members, it can also be important to have a conversation about racism, discrimination, marginalization and other systemic and contextual factors that can negatively impact a youngster's well-being and contribute to self-harm behavior (74). Because it can be difficult to directly impact some of the systemic forces directly, it can be particularly important to mobilize the youth's and the family's resiliency factors. For example, family therapy can help parents share survival strategies and effectively advocate for their kids.

## Enhancement of the family therapy component

CIFFTA's family work typically consisted of (1) developing a systemic conceptualization of the presenting problem(s) and family functioning, (2) engaging and joining with family members, some of whom may be reluctant to participate in treatment, (3) identifying and reducing conflict and maladaptive family processes, and (4) mobilizing protective, supportive and healing family processes. By helping the family find new solutions, the therapist can increase family flexibility. CIFFTA therapists observe and shape *in vivo* family interactions that support or constrain the adjustments the family must make to reduce self-harm. Family enactments allow the therapist to identify and modify key relational patterns.

Developing a systemic conceptualization meant identifying the ways in which *larger community and societal forces* could negatively impact not only the adolescent but also the family that might otherwise buffer the youth from risk (75). Economic stress, work related stress, neighborhood stress, must all be on the radar because they could easily reduce parent time available in the home, and increase family stress and the youth's isolation. Further, during difficult times it was not unusual for the youth to feel they could be a burden on the family (76). This sense of burden could fuel self-harm ideation. In some situations, anti-immigrant dialogues and culture-related worldviews and values could also negatively impact the family and constrain change. In the domains of symptom recognition, problem labeling, help-seeking behavior, and therapy processes, variations can often be linked to ethnicity, race and worldviews (77). The therapist's ability to explore and open the door to discussion of these broader forces and how they may impact risk for self-harm is key to culturally and contextually informed work. Although therapists cannot always modify the systemic pressures and stressors, they can help to modify the impact on the family and the family-level mediators (e.g., marginalization and isolation of youth who feel they are a burden) to reduce self-harm.

As we began to *engage and join family members*, it became obvious how self-harm was a particularly alarming and intimidating presenting problem, and that family members often responded in unexpected ways. When a family learned that their child or adolescent was reporting self-harm behavior, they sometimes reacted by minimizing or even fully denying the risk posed by the adolescent's behavior. This could be fueled by a sense of hopelessness and helplessness (35). How a therapist handled this communication could impact engagement and joining. It is a mistake to focus on the minimization or denial without appreciating the hopelessness and despair that may be fueling it. When families say that it may just be adolescent attention-seeking or manipulative behavior, it is often a way of coping with the despair that comes from having a child that has the potential to take their own life. Therapists must do their best to engage all family members and observe their interactions to properly identify the unique family processes that may trigger self-harm, reduce help-seeking behavior, and constrain change.

CIFFTA has always worked to reduce conflict, isolation, and disengagement while increasing the *family's acceptance, validation, and support of the adolescent*. Intergenerational differences in acculturation or parent-youth acculturation gap (78) have been identified as associated with Latine female self-harm and suicide behavior. The conflicts that come from this process are addressed in family therapy, first by normalizing it and then by working it through. Normalization means that we show how common it is for these differences to emerge as youth acculturate more rapidly and fully in a new host culture. It is not a decision to reject the culture of origin or the values and history that families hold dear. Once the anger and frustration are reduced, the family can process the benefits of integrating multiple cultures and perspectives. Another area that Latine families must be more sensitive to is when a Latine youth is feeling the stress of being transnational and feeling alienated, marginalized and lacking connection due to their Latine identity (78). While this is addressed in individual therapy with the adolescent, family work can also focus on the value of support and validation from family members. One of the strengths of family therapy is the use of enactments to assess and then shape family relational patterns and interactions to include more support and better communication.

As families learned about support and validation, they also learned that validation does not mean that they must agree with all the adolescent's positions or views. It is more that youth should be allowed to have a perspective and that they can be accepted as people aside from their preferred behaviors. Support, acceptance and validation became particularly important in families with LGBTQ+ youth (54). This was especially true when family members were first learning about the youth's sexual orientation or gender identity. Family rejection can be particularly painful for these youth and rejection can be a trigger to self-harm behavior (54). Cultural factors and traditional worldviews can make it harder for some families to accept their youth when they break away from norms or expectations and this can be particularly powerful in the families of LGBTQ+ youth. Interestingly, factors that are often considered protective such as familism (i.e., a focus on the well-being of the family) and religiosity, can make acceptance more difficult. Many of these processes can add to the stigma and avoidance reaction. Therapists cannot be thrown off course by this stance and must avoid blaming the family. It takes special skill for therapists to allow the struggle parents are having with extended family or outside support systems to emerge without judgement, and to work through how to juggle the tensions.

### Supporting strong family processes around technology enhancements

When technology is being used, it is helpful to use it in a way that supports the types of healthy family interactions endorsed by the CIFTTA model. Parents were asked to watch psychoeducational videos on subjects of importance to their adolescent's first (e.g., substance use, depression, self-harm), so they could be informed on the topic and prepared to provide guidance on the issue prior to watching the video with their child. The importance of this strategy emerged in our prior research with CIFTTA when Latine parents reported that one of the major obstacles to providing guidance on safe sex and contraceptive use was a lack of information on the topics (79). This finding led our team to understand the importance of giving parents information, commonly used words and terminology, and tools that enabled them to feel confident to *provide leadership* on issues critically important to the adolescent. When parents can provide guidance, it has a more powerful and long-lasting impact than when the therapist provides the guidance.

## Guidelines for delivering CIFFTA via telehealth services

Changes in the delivery of mental health services following COVID meant an increase in the use of telehealth services (44). As part of this work, guidelines were established to address challenges to delivering the CIFFTA intervention remotely via Zoom. The guidelines focus on using technology effectively and offer best practices for delivering video sessions. The guidelines are also helpful during supervision meetings to shape effective practice.

One important topic was ensuring safety during virtual treatment sessions. Guidelines emphasized the need to develop an emergency plan with each family at the start of treatment that included dialing 911 if the clinician felt that the youth or caregiver were in danger to themselves or others. It was emphasized that a caregiver should be present in the home at the time of sessions and clinicians should have the contact information available for the caregiver as well as additional emergency contacts for the youth.

A second area was structuring the environment and facilitating privacy. During the first CIFFTA Telehealth session with the family using Zoom, the clinicians discussed the telehealth setup and privacy with all members of the family together. It was important to prepare for difficulties that could be encountered during delivery of the CIFFTA components (family, individual, and psychoeducational sessions). Individual and some psychoeducation sessions are for the youth alone while parental subsystem sessions and certain psychoeducation sessions are for caregivers alone, thus the issue of privacy was important to discuss and plan for ahead of time. In addition to the usual planning to avoid distractions (e.g., phones, dogs, neighbors, TV, cooking) it was also important to plan the use of space so that sessions could have some level of privacy (even if it meant having the session sitting alone in a parked and accessing the virtual platform via the phone). The concern was that the failure to achieve privacy could severely limit the content that a family member would be willing to share during a virtual session. Over time, this could mean that some CIFFTA components could be underutilized. Some of these structuring tasks important to telehealth delivery are similar to what may be required when conducting homebased treatment (80).

CIFFTA psychoeducational sessions with youth and/or parents may be particularly challenging because the psychoeducation sessions are didactic in nature, and it can be harder to maintain their attention on the phone or computer. Guidelines included emailing the content of the module to youth or caregivers prior to the psychoeducational session so that they could have the material to reference back to. If they had access to a printer, they could also print the materials. The use of the Zoom “share screen” tool and the whiteboard feature with families and especially with adolescents, was helpful for these sessions. Guiding them through the “annotation” features on Zoom helped engage clients since they could interact directly with the material by highlighting, writing, or drawing (e.g., emotion regulation skills). Finally, during the CIFFTA psychoeducation sessions it was important for clinicians to actively engage with the youth and/or caregivers. Given that a considerable amount of information was being shared, regularly checking in with them and using the discussion questions built into the psychoeducational module helped maintain the interest of adolescents or caregivers. In summary, while CIFFTA can be effectively delivered through technology, it was not always the preferred method and was the exception rather than the rule. As mentioned above, the telehealth delivery of CIFFTA presented certain challenges that could significantly impact the quality and fidelity of the treatment, impacting the outcomes for the youth and their family. Each case needed to be assessed individually to determine the effectiveness of telehealth as a delivery method for CIFFTA.

## A case study exemplifying the use of CIFFTA components to reduce youth self-harm

As we have reported elsewhere (81), our enhanced CIFFTA was highly acceptable to the youth and families. On average, CIFFTA families received 24 total sessions, comprised of 8.1 individual therapy sessions, 9.2 family therapy sessions, and 6.2 psychoeducational sessions. The total number of sessions and the distribution in the three CIFFTA components is consistent with the way CIFFTA was designed to be delivered (35). Given concerns that the Latine population underutilize services and tend toward premature termination, this profile of service utilization is quite promising. In this section we share a case study that shows the CIFFTA components in action and creating synergy to impact self-harm, emotion regulation, and family connectedness. In this profile the name of the youth and some facts were changed (using a composite of case studies) to protect privacy.

Erika was a 15-year-old female born in the U.S. while her parents and an older brother (age 19) were born in South America. The family had been in the U.S. for 17 years. Erika was referred to our program because she was cutting herself over the past year and more recently, she was attempting to conceal the behavior by cutting areas of her body that were less visible. She claimed she did not think about ending her life but simultaneously stated that she understood why some people suicide to end the distress. Erika shared that she cut herself when she was feeling *highly stressed and emotionally confused*. She also often reported feeling sad. When asked about her gender identity and sexual orientation, Erika reported feeling female and had begun to question whether she was attracted to both females as well as males. She said she was not yet sure. Erika's parents were willing to participate in treatment although they were not comfortable with the idea of therapy. They felt that the main problem was that Erika was too dramatic and exaggerated her problems. The family described themselves as *traditiona*l and as maintaining their Latine values and customs. They stated that Erika seemed to rebel against those customs and traditions.

### Tailoring treatment using the CIFFTA psychoeducational component

Based on the CIFFTA tailoring profile the modules most relevant for Erika and her family were the Self-Harm and Depression modules (delivered to the family together), and the Distress Tolerance and the Emotion Regulation modules (delivered to Erika alone). The Parenting module (delivered to the parents alone) is delivered to all families and was the module that the therapist delivered first. Although the Acculturation module was considered, the use of 6 modules might have been overwhelming for a family in treatment. The family agreed that the 5 modules might be useful given their perspectives on the presenting problems. Having parents who were willing to participate in treatment and a supportive brother were considered protective factors.

Erika reported difficulty with controlling her emotions, and when they became overwhelming, she engaged in cutting to reduce their intensity. This discussion led to the introduction of the Distress Tolerance and Emotion Regulation modules as ways to help her manage these moments. The Distress Tolerance module focused on developing a tailored plan to use in the moments when emotions become unmanageable. Erika found the module helpful because it allowed her to try different strategies until she found the ones that worked best for her. Erika found that strategies related to the sense of touch worked well. She used stress balls and ice cubes on her arms to get her through the most difficult periods. The Emotion Regulation module was introduced to help Erika identify, name, and accept emotions that she experienced. As Erika learned skills that she found helpful in regulating her emotions, this allowed her to manage her reactions, utilize distress tolerance coping, and reduce the urge to self-harm. Some of the module sessions were delivered in virtual fashion. A short engaging video was used during the session to introduce the skills concepts to Erika. The therapist used the Zoom *share screen* tool and the whiteboard feature. Erika enjoyed interacting with the technology and adding notes as the discussion with the therapist took place. Specifically, she used the *annotation* features to highlight, write, and draw her own *take* on the topic and day to day examples.

The Self-Harm and Depression modules were delivered to the family conjointly because all family members were aware of the self-harming behaviors and Erika's symptoms. The Self-Harm module provided the definition and rates of self-harm and described interpersonal and intrapersonal triggers to the behaviors and ways to respond to the triggers. The family responded well to the module as it reduced the stigma around self-harm and helped them to understand the kinds of stressors that are linked to the behavior. When family members learned to manage their anxiety and understood that the behavior was a result of distress and not merely manipulative or attention-seeking behavior, they were better able to respond effectively. The Depression module helped the youth and family to detect and understand how sadness and depression manifest themselves in daily life and how the family can be supportive. It was important for them to understand that depression and sadness can easily be masked by more dramatic self-harm behavior. The Parenting module provided to the parents alone, shared information on Erika's developmental stage, effective communication strategies, how to detect and react to sadness and self-harm behavior, and how to validate her experiences. The therapist encouraged the mother and father to share their values and beliefs around family, culture, and parenting but also understand that it is normative for kids not to share all of these values and beliefs. Parents were taught that having a conversation about self-harm behavior did not increase the likelihood of the behavior.

### Individual therapy sessions with Erika

Early individual sessions with Erika focused on getting to know her interests and concerns and increasing her motivation to use treatment to reduce her self-harm behaviors. Erika liked talking about her interest in soccer. Erika admitted that she became frustrated quickly and unexpectedly and that she wanted help with this so that her reactions did not hurt her relationships with friends and family. Individual sessions were also the place where Erika was able to explore her feelings about her sexual orientation without judgement. She was not particularly interested in sexual relationships yet, and she did not feel this was related to her emotional reactions, so she did not want to prioritize this work. Should she have been ready to address this more fully, CIFFTA provides therapists with psychoeducational material that helps them promote a healthy discussion on the topic.

Mid and late phase individual sessions with Erika focused first on monitoring her self-harm behaviors and ensuring that these behaviors were not deteriorating. One reason to include individual sessions is that youth may be more honest about this risk in individual sessions as compared to family sessions. The goal is to reach a point when all these issues can be discussed openly and honestly in family sessions. Individual sessions were also used to generalize the material from the Distress Tolerance, Emotion Regulation, Depression, and Self-Harm modules. Generalization work focused on identifying the life situations where the skills could be used and any adjustments needed to be more effective. As the therapist monitored Erika's emotional dysregulation and urges to self-harm, a great deal of the discussion revolved around which skills could address the problem, what barriers blocked the use of skills, and reinforcing Erika for the proper and successful use of skills when she used them. Diary cards were effective in documenting urges, triggers, and barriers to the use of skills. Halfway through treatment, Erika was no longer cutting.

After the family completed the Self-Harm module, the therapist used individual sessions to provide Erika with a space to share her emotions, and her feelings of rejection and not being understood by her family. The therapist worked with Erika to shape the communications she had with her parents during family sessions and coached her to be successful in those family sessions. Erika began to experience the parental behaviors as protection and caring rather than as controlling behavior and this led her to relate to them in a healthier way. Because the therapist was working with parents separately using the parenting module, this key process was being addressed from multiple angles, creating synergy toward change.

### Family therapy sessions with Erika

Early family work focused on engaging and joining all family members. It was important to engage the older brother into therapy as siblings are often underutilized in family treatment. Siblings can be a powerful element in treatment because they have shared many experiences amongst each other. The therapist took the time to hear about the family's immigration and acculturation experiences and to validate the family's reluctance to share personal information with a stranger. This meant working to understand the parents' worldview regarding mental health treatment. The family's reluctance to *air dirty laundry in public* was one of the main reasons that they were reluctant to seek help for Erika sooner. It is difficult to expose personal family material that family members may not be proud of. Part of the reluctance was also a tendency by some parents to minimize the significance of self-harming behavior. This response is partly defensive given how helpless and hopeless they felt. The stigma around emotional struggles and mental health treatment was powerful but was successfully reduced through the modules and family sessions.

Given that acculturation was a topic of conversation, it helped to describe therapy as coaching or fine-tuning parenting in a country with such different values and customs. If the therapist can convey that family values and perspectives are an an important part of therapy, parents are more likely to feel respected, understood, and open to participating in therapy. The culture-related conflicts that emerged in the family were normalized as something that occurs often in first-generation immigrant families and not simply dysfunction in a family.

Later family sessions focused on identifying family interactions and patterns that needed to be modified. Family *enactments* during which the family members show the therapist their typical and usual relationship patterns, allowed the therapist to see the repetitive behaviors that shut down or trigger another family member. For example, when family members communicated in a therapy session that the youth's distress was exaggerated to get attention or to manipulate others, the therapist could highlight the *in vivo* results of this invalidating communication (e.g., Erika shutting down in session). The therapist shaped the interactions, moving them in the direction of support and validation. The therapist used the sessions to *generalize* what was learned in the modules on self-harm and depression, to these in-session moments. This family work and the concurrent individual work with the adolescents focused on similar issues so CIFFTA worked on achieving key changes from both sides of the circular relationships. The family sessions were the arena in which Erika was able to practice her emotion regulation skills, particularly during heated discussions. The therapist helped family members identify and reinforce the good work Erika was doing in skills sessions and in individual sessions.

Erika and her family received treatment for approximately 20 weeks and attended 28 sessions. Overall, they received 12 family sessions, 8 individual sessions, and 8 psychoeducational module sessions. At termination, Erika reported that she had not engaged in self-harm since week 8 of treatment. The family improved their communication, and the mother and father had gained a better understanding of Erika's self-harming behavior. These important changes were documented using the Deliberate Self-Harm Inventory–Youth Version [DSHI-Y; (82)] and the Family Connectedness (FC) scale (83). Sibling sessions between Erika and her brother solidified his support for her and allowed them to share their perspectives on various stressors they faced together, including acculturation stress. Erika experienced a significant increase in validation from her entire family.

## Discussion

Research has supported CIFFTA's efficacy as a flexible, culturally informed and manualized multi-component treatment, but CIFFTA had never been used to treat youth showing suicide risk and/or self-harm behavior. The work reported here took advantage of CIFFTA's flexible and modular approach to incorporate new content and processes to address the unique clinical and cultural characteristics of Latine, Black, and LGBTQ+ youth reporting self-harming behavior and their families.

The work sought to address a shortage of manualized treatments that have cultural considerations and material at their core. Too often, talented clinicians must combine on their own what they have learned about cultural sensitivity, family mechanisms of change, and self-harm. Elsewhere we have made the argument that to be a good candidate for research and to be replicable, a manualized treatment should integrate what is known about the interaction of specific presenting problems, mechanisms of change, and cultural material (34, 35). The integrative work reported here sought to meet this need and to create a manualized intervention that could lend itself to a clinical trial (81). To ensure that this evidence-based and manualized treatment can be tested and later replicated, we documented the new psychoeducational modules, the generalization work, and the individual and family processes that are key to individual and family treatment for the presenting problem of self-harm.

At the individual adolescent level, the development work led to a strong array of interventions including Motivational Interviewing, skills for youth, use of diary cards, and a focus on how to prepare the youth for more adaptive family interactions. Many of these dimensions (e.g., DBT skills and a significant amount of work with the adolescent) were among the factors that Brent et al. (29), found to be helpful in the treatment of self-harm and suicide risk in youth. Although we hypothesize that family factors are powerful predictors of success, we strongly believe that the motivation to change and many of the steps toward change must also come from the youth themselves.

At the level of the family, there are many factors that keep individual family members from engaging in and remaining in treatment. Engagement into treatment can be disrupted by a worldview that conveys that treatment is for people with more severe mental illness and that stigmatizes self-harm. When families feel helpless to address such a powerful topic, there may be a tendency to deny or minimize its significance. The CIFFTA resources provided to therapists in the form of the self-harm module and in the form of family interventions aim to help them address these barriers to treatment. By being prepared to directly address the cultural and clinical factors that can be barriers to treatment, the therapist can be more successful at engaging family members and bringing about relational change. Further, by manualizing these approaches and strategies, the treatment can be more easily tested and replicated.

Finally, we have explored the integration of technology into treatment because it promises to address some of the challenges and barriers to service delivery. Our experience is that virtual sessions and technology assisted services were not for everyone. However, for many clients and for some specific components such as psychoeducational work, technology may be promising in its ability to have clients work during the week prior to in person sessions. The therapist and the family together must assess the value of technology assisted services, component by component, and thoughtfully decide how they can best be used.

These enhancements were consistent with CIFFTA's philosophy that a therapy should integrate the individual and family underlying mechanisms of action (e.g., the enhancement of motivation, reduction of dysregulation, and improved family functioning) with the unique stressors and life experiences that are confronted by the youth and families we seek to serve. It is difficult to argue that a therapy is ecologically valid (37) if it fails to acknowledge and address the role that stressors such as racism, discrimination, and anti-immigrant messages can have in undermining a youngster's and a family's well-being. By integrating this material right into the manual and modules, the CIFFTA therapist is always prepared with tools to address these critically important issues. We believe it is less effective to place the onus of adaptation on the therapist while also requiring that they remain faithful to the principles of the evidence-based treatment. CIFFTA's treatment enhancement effort includes tools that we think can make treatment easier to implement/deliver (e.g., due to the modular format and psychoeducational material), more meaningful (e.g., due to inclusion of daily stressors), engaging (e.g., due to multimedia material that can be used even between sessions) and powerful (e.g., because it includes well-established family and individual components).

There are important limitations to the work reported here. Some refinements to CIFFTA were not planned but were a response to the COVID-19 pandemic and subsequent disruptions to in-person treatment. A second limitation of this manuscript is that it includes only participation and service utilization. We report elsewhere (81) the outcomes of the randomized trial that investigates the youth and family impact of this technology assisted CIFFTA.

## Data Availability

The datasets presented in this article are not readily available because not applicable. Requests to access the datasets should be directed to mmena@miami.edu.
